# Essential Oil of *Cannabis sativa* L: Comparison of Yield and Chemical Composition of 11 Hemp Genotypes

**DOI:** 10.3390/molecules26134080

**Published:** 2021-07-03

**Authors:** Ylenia Pieracci, Roberta Ascrizzi, Valentina Terreni, Luisa Pistelli, Guido Flamini, Laura Bassolino, Flavia Fulvio, Massimo Montanari, Roberta Paris

**Affiliations:** 1 Department of Pharmacy, University of Pisa, Via Bonanno 6, 56126 Pisa, Italy; ylenia.pieracci@phd.unipi.it (Y.P.); vale.terreni@gmail.com (V.T.); luisa.pistelli@unipi.it (L.P.); 2 Interdepartmental Research Center “Nutraceuticals and Food for Health” (NUTRAFOOD), University of Pisa, Via del Borghetto 80, 56124 Pisa, Italy; 3 CREA—Research Center for Cereal and Industrial Crops, Via di Corticella 133, 40128 Bologna, Italy; laura.bassolino@crea.gov.it (L.B.); flavia.fulvio@crea.gov.it (F.F.); massimo.montanari@crea.gov.it (M.M.); roberta.paris@crea.gov.it (R.P.); 4 Department of Sciences of Agriculture, Food Natural Resources and Engineering, University of Foggia, Via Napoli 25, 71122 Foggia, Italy

**Keywords:** monoecious, dioecious, by-products, monoterpenes, sesquiterpenes, cannabinoids, flowering behaviour, cannabidiol

## Abstract

*Cannabis sativa* L. is an annual species cultivated since antiquity for different purposes. While, in the past, hemp inflorescences were considered crop residues, at present, they are regarded as valuable raw materials with different applications, among which extraction of the essential oil (EO) has gained increasing interest in many fields. The aim of the present study is the evaluation of the yield and the chemical composition of the EO obtained by hydrodistillation from eleven hemp genotypes, cultivated in the same location for two consecutive growing seasons. The composition of the EOs was analyzed by GC–MS, and then subjected to multivariate statistical analysis. Sesquiterpenes represented the main class of compounds in all the EOs, both in their hydrocarbon and oxygenated forms, with relative abundances ranging from 47.1 to 78.5%; the only exception was the Felina 32 sample collected in 2019, in which cannabinoids predominated. Cannabinoids were the second most abundant class of compounds, of which cannabidiol was the main one, with relative abundances between 11.8 and 51.5%. The statistical distribution of the samples, performed on the complete chemical composition of the EOs, evidenced a partition based on the year of cultivation, rather than on the genotype, with the exception of Uso-31. Regarding the extraction yield, a significant variation was evidenced among both the genotypes and the years of cultivation.

## 1. Introduction

*Cannabis sativa* L. is an annual herb belonging to the *Cannabaceae* family, which has been cultivated since antiquity as a source of fiber, seed oil, food, and medicine, as well as for recreational and religious purposes [1].

It has evolved as a dioecious species, with female and male flowers on different individuals, but selection processes have led to the development of monoecious genotypes that bear male and female flowers on the same individual. Thus, depending on the intended use, the morphology of the plants varies significantly between genotypes, in terms of height, biomass, and seed yield [1,2].

*Cannabis* female inflorescences and leaves are covered in glandular trichomes, which are considered as bio-factories of phytochemicals [3] due to their ability to synthesize and store different secondary metabolites, of which phytocannabinoids are the best known and studied [4]. On the basis of their cannabinoid content, in particular of their cannabidiol (CBD)/tetrahydrocannabinol (THC) ratio, *Cannabis sativa* L. genotypes are divided into five distinguished chemical phenotypes: (i) chemotype I, or drug-type (the predominant cannabinoid is THC); (ii) chemotype II, or intermediate-type (the predominant cannabinoids are CBD and THC); (iii) chemotype III or fiber-type (the predominant cannabinoid is CBD); chemotype IV (with a prevalence of cannabigerol, CBG) and chemotype V, classifying materials with undetectable amounts of any cannabinoid [5,6].

Since 2001, when the EC No. 2860/2000 entered into force, the European Union authorized the cultivation of hemp complying with the 0.2% *w/w* Δ^9^-THC threshold [7]. As a consequence, hemp cultivation for fiber and seed production was resumed, and more attention was paid to agro-industrial waste of the hemp chain, among which inflorescences, as valuable sources of bio-active molecules to feed pharmaceutical, cosmeceutical, and manufacture industry, in the perspective of the sustainable circular economy. In recent years, the extraction of hemp essential oil (EO) has gained increasing interest as a value-added product [8], thanks to its various fields of application [9]. Hemp EO showed its best outcomes as an environmentally friendly insecticide against aphids, housefly populations, and mosquitoes [10], as a noteworthy toxic effect against *Aedes albopictus* is reported. Moreover, it exerts a good toxic activity towards the snail *Physella acuta*, an intermediate host of nematodes and trematodes human parasites, as well as being a common disease for rice fields [11]. In the agricultural field, the hemp EO exhibits a strong allelopathic activity against invasive weed germination, as well as seedling growth [12]. Interestingly, EOs were reported to be effective against dermatophytes species, thus exerting a role in preventing skin disorders [13]. Moreover, its use as a beverage flavoring agent was reported [9].

Essential oil from *Cannabis* is a complex mixture of volatile compounds made up of more than 100 terpenes and terpenoids (the oxygen-containing terpenes), known as the major contributors of the peculiar aromatic profile of different *Cannabis* strains [14,15,16]. Monoterpenes (10 C) and sesquiterpenes (15 C) constitute the largest content of the hemp essential oil, in both their hydrocarbon and oxygenated forms [17,18], followed by diterpenes (20 C). Almost every compound identified in the EO has its own characteristic fragrance, and their combination is responsible for the unique aromatic bouquet of different strains [19], which can influence consumers’ preferences: Generally, hemp varieties with high percentages of monoterpenes are considered more pleasant than those with high percentages of sesquiterpenes [20]. Several factors, such as genotype, flowering behavior (dioecy or monoecy), cultivation technique, plant density, stage of development at harvest, material processing, and storage conditions can influence the composition of hemp EO (chemical profile) and its extraction yield. [19,21,22,23,24]. Monoterpenes could be present in higher quantities in the fresh material, while drying and storage could determine their loss, leading to the increment of the relative portion of sesquiterpenes. The environmental [8,21] and the weather conditions seem to be an important factor for both the EO composition and yield; indeed, dry conditions between flowering stage and seed maturity can prevent trichomes damage and EO yield losses [22].

All these factors affecting the yield and chemical composition of hemp essential oils, as well as the lack of standardization of growing and operating conditions, make it hard to compare the chemical composition of the essential oils extracted from different *Cannabis sativa* L. genotypes in diverse studies.

The present study aimed to evaluate the EO yield and the volatile profile of 11 different hemp genotypes, both monoecious and dioecious, belonging to three chemotypes (III–V), and the differences that occurred during two years of cultivation, in order to promote their employment as value-added by-products based on their peculiar characteristics.

## 2. Results and Discussions

### 2.1. Plant Material Features

The cultivation in open field for two consecutive years included five monoecious (Uso-31, Carmaleonte, Codimono, Futura 75, Felina 32) and six dioecious (Bernabeo, Carmagnola, CS, Fibranova, Fibrante, Eletta Campana) European industrial hemp genotypes. The main characteristics of plant material, including chemotypes, flower type, and flowering time, are reported in Table 1.

Sowing was performed on 19 April 2019 and 23 April 2020, with a 25 cm distance between rows on randomized blocks with three repetitions, and a plot size of 20 m^2^.

The harvest date differed according to the flower behavior and flowering time of each genotype. Generally, monoecious and early-flowering strains were harvested earlier than dioecious and late-flowering genotypes in both years (Table 1).

The comparison between plant heights showed differences between the two years, with higher plants obtained in 2020. The same trend was observed when comparing plant collar diameters. Plant density, defined as number of plants/m^2^, and dried inflorescences yield/m^2^ were significantly lower in 2020, while dried inflorescences yield calculated for single plant was by far higher in 2020 than in 2019. These differences in plant growth in the two years of cultivation could be due to the lack of rainfall in the pre- and post-emergence period of 2020, compared to that of 2019, which could have caused a reduced germination rate and, therefore, a lower plant density; conversely, 2019 was very rainy in May–June, favoring seed germination and following plant density. This rainfall trend was inverted in June; indeed, the rainy period (July–August) in 2020 could have allowed for a biomass production greater than that observed in 2019, as is clearly shown in Table 1. Finally, September 2019 was rainier than 2020, and this could have influenced the EOs’ yield.

### 2.2. Essential Oil Compositions and Yields

The complete compositions and the yields of the essential oils obtained from the dried inflorescences of 11 genotypes of *Cannabis sativa* L., cultivated by CREA-CI (Italy) in an experimental farm located in Rovigo and harvested during the years 2019 and 2020, are reported in Table 2 and Table 3, respectively. In total, 116 compounds were identified, representing 90.6–99.4% of the total composition.

All the EOs, with the exception of Felina 32-2019, were characterized by a predominance of sesquiterpenes, in both their hydrocarbon and oxygenated forms, ranging from 47.1% in Carmagnola-2020 (Table 3) to 78.5% in Fibrante-2019 (Table 2). As reported in Table 4, both the hydrocarbon and oxygenated sesquiterpenes presented significant differences in all the EO compositions as a function of the genotype, the year of cultivation, and their interaction.

In 2019 EOs, sesquiterpene hydrocarbons exhibited a significantly higher relative concentration in Futura 75, followed by Fibrante and Carmagnola. Lower relative abundances were, instead, found in Bernabeo, Eletta Campana, and Felina 32 (Table 2). In 2020 samples, instead, this class of compounds was significantly more abundant in Carmaleonte, Codimono, Eletta Campana, Fibranova, and Futura 75, while their lowest presence was detected in Uso-31 (Table 3). Furthermore, sesquiterpene hydrocarbons were significantly higher in 2020 than in 2019 for each analyzed hemp genotype, except for Carmagnola and Uso-31. Among this chemical class, the main volatile compounds were β-caryophyllene and α-humulene, in accordance with Ascrizzi et al. [9], Vuerich et al. [18], and Menghini et al., 2021 [25], who reported these constituents as being typical of hemp varieties.

Regarding the oxygenated sesquiterpenes, among the 2019 EOs, Carmaleonte and Uso-31 presented higher relative abundances (59.6 and 60.9%, respectively), whilst the lowest was exhibited by Felina 32 (30.9%). Conversely to the hydrocarbon forms, oxygenated sesquiterpenes were significantly more abundant in all the 2019 samples compared to those of 2020. These secondary metabolites are degradation products deriving from the oxidation of the corresponding terpenes due to air exposure (i.e., during prolonged storage), and are considered responsible for the antioxidant activity of many EOs [18,26]. Moreover, higher oxygenated compounds’ relative abundances reflect more favorable growth conditions, as was also confirmed by the higher plant density detected in the 2019 samples. The analyzed samples presented caryophyllene oxide and humulene oxide II as the main components of this chemical class, which were also reported as the main epoxides found in the hemp varieties analyzed by Micalizzi et al. [27]. Furthermore, 14-hydroxy-9-*epi-(E)-*caryophyllene, reported by Ascrizzi at al. [8], was present in low relative abundances.

The amount of selinene derivative compounds (α-selinene, β-selinene, selina-3,7(11)-diene, and selin-6-en-4-ol) in 2019 and 2020 is notable, as this is a common occurrence in hemp EOs; their presence was more consistent in Futura 75, Eletta Campana, and CS, as already reported in the literature [10,25,27]. Bernabeo EOs in both years were characterized by the presence of higher percentages of *neo*intermedeol, α-bulnesene, and juniper camphor; Fibrante EOs by *trans*-α-bergamotene and (*E*)-β-farnesene.

Monoterpenes were poorly represented in all the samples, with no distinction between monoecious and dioecious varieties, but with differences induced by the year of collection and the genotype. They accounted for up to 9.3% in Carmagnola 2020 (Table 3). Overall, 9 hydrocarbons and 14 oxygenated monoterpenes were detected: among the former, α-pinene, β-pinene, and myrcene were the most representative, whilst in the latter, no compounds were revealed in appreciable relative abundances. Among the 2019 EOs, monoterpene hydrocarbons were significantly higher in Carmagnola and Fibrante (2.0 and 2.1%, respectively), and the latter presented a significantly higher content of oxygenated monoterpenes as well (1.3%). Fibrante-2019 also differed from the other genotypes in terms of its higher relative abundance in limonene (0.5%), which reached 0.8% in 2020. In the 2020 samples, hydrocarbon forms were found to be more abundant in Carmagnola (1.8%), while the oxygenated ones in CS (2.3%). However, with the exception of Codimono, 2020 samples were characterized by a significantly higher relative abundance of both hydrocarbon and oxygenated monoterpenes.

The amount of the compounds belonging to this class of secondary metabolites is very different from those reported by Bertoli et al. [21], Benelli et al. [10] and Nissen et al. [28], in which the main detected chemical class was represented by monoterpenes, with significant differences between monoecious and dioecious genotypes; in these papers, however, the starting material consisted of the fresh inflorescences, rather than the dried ones. Assuming that the monoterpene content decreased in the drying process and storage [26,27,29,30], the predominance of sesquiterpenes in the examined EOs might be due, at least in part, to the drying process, which might have induced some changes in the chemical composition of the starting material, such as (i) evaporation of the low boiling-point compounds [27], and (ii) the induction of oxidative reactions, as in the conversion of β-caryophyllene in caryophyllene oxide, and α-humulene in humulene oxide II [30]. Nevertheless, few of the literature studies used the dried hemp inflorescences for the collection and characterization of the EOs.

Finally, cannabinoids were the second relevant class of compounds in all the EOs, excluding that of Felina 32-2019, in which they were the main chemical group, accounting for the 53.4% of the total (Table 2). The detected cannabinoids were cannabidiol, cannabichromene, and Δ^9^-tetrahydrocannabinol, but the first was the most abundant, with relative percentages ranging from 11.8 to 51.5% in Uso-31-2019 and Felina 32-2019, respectively (Table 2). In 2020 samples, these metabolites were detected in a greater relative amount in Carmagnola (37.7%) than in Bernabeo (15.0%, the lowest). In general, cannabinoids were meaningfully higher in the 2020 samples, except for Bernabeo and Felina 32, but according to the two-way ANOVA (Table 4), no significant differences were present between the two years of cultivation for this chemical class, which, on the contrary, showed significant differences among the genotypes. As for the sesquiterpenes, the predominance of cannabinoids in the EOs might be imputable to the drying process, during which the decarboxylation of cannabinoid acids into their relative volatile forms takes place [30].

Nevertheless, all the detected classes of compound, excluding the already mentioned cannabinoids, presented significant differences as a function of genotype (G), year of cultivation (Y), and genotype–year interaction (G × Y), as evidenced by the two-way ANOVA (Table 4), which was also conducted on the chemical compounds, selected by the SIMPER test, which contributed to at least 1.00% of the dissimilarities in the composition between 2019 and 2020 EO samples. The statistical analysis evidenced that 16 compounds were responsible for an overall dissimilarity contribution of 32.94%, and only seven accounted for a total dissimilarity contribution above 55%. They comprised (i) the two main sesquiterpene hydrocarbons, β-caryophyllene and α-humulene, (ii) four oxygenated sesquiterpenes, caryophyllene oxide, 14-hydroxy-9-*epi-(E)-*caryophyllene, and the two isomers of caryophylla-4(14),8(15)-dien-5-ol, and (iii) one cannabinoid, cannabidiol: all the selected chemicals had significant differences based on the genotypes, year of cultivation, and their interaction.

The essential oil extraction yield ranged from 0.03 to 0.12% *w/w* for the samples collected in 2019, and from 0.03 to 0.23% *w/w* for the 2020 samples, where the highest yield was comparable to those already published in the literature [20]. A statistically significant difference in percentage was found among genotypes, year of cultivation, and the interaction between genotype and year (Table 4). Regarding the dependence of the EO extraction yield on the hemp genotype, several studies are present in the literature [18,19,22,28,31], while no references are available for its dependence on the year of cultivation.

For the 2019 samples, the significantly highest EO extraction yield was obtained by Futura 75 (0.12%), followed by Carmagnola (0.09%) and Eletta Campana (0.09%), while the lowest (0.03%) from Bernabeo, Felina 32 and Uso-31. Regarding the 2020 samples, instead, Codimono, followed by Eletta Campana > CS > Fibrante (0.23% > 0.20% > 0.20% > 0.16%, respectively), were the most productive cultivars, whilst Uso-31 and Bernabeo were the least productive ones (0.03% and 0.05%, respectively). For Uso-31 (chemotype V), whose inflorescences are characterized by a negligible level of cannabinoids, the lowest EO extraction yield was found irrespective of the year. A positive correlation between the accumulation of total cannabinoids and total terpenes, both synthesized in glandular trichomes [22], was already reported [32,33,34], and explains the low EO yield obtained for Uso-31. Considering both the years of cultivation, all the 2020 samples were characterized by a significantly higher EO extraction yield than the 2019 ones (Table 2 and Table 3), and this could be at least partly due to the different meteorological conditions occurring in the two subsequent growing seasons, particularly regarding rainfalls and average temperature (Table 5). Higher temperatures might, indeed, cause a higher spontaneous evaporation degree of the EO from the plant trichomes.

### 2.3. Multivariate Statistical Analysis

**Figure 1:**
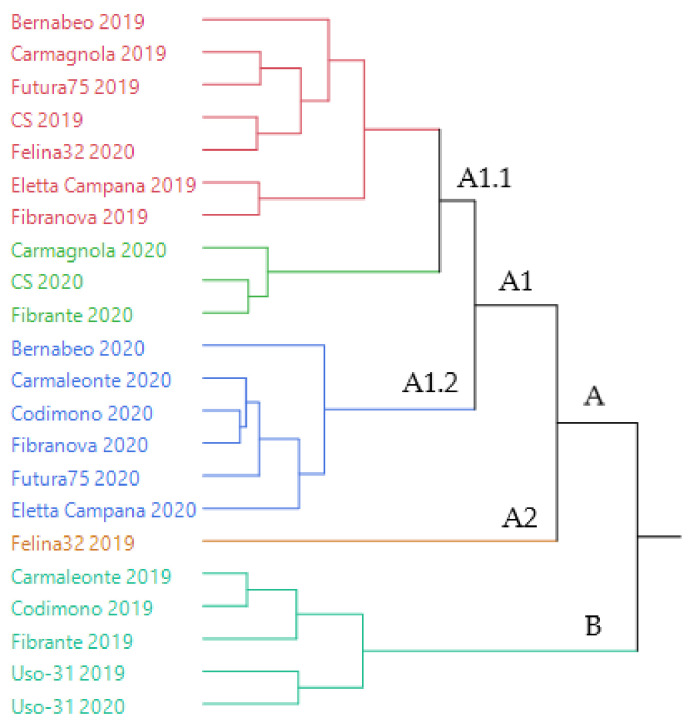
Dendrogram obtained with the hierarchical cluster analysis (HCA) performed on the complete composition of the essential oils extracted from all the hemp samples.

The dendrogram of the hierarchical cluster analysis (HCA) performed on the complete compositions of the EOs, extracted from all the samples of hemp of both years of cultivation, is reported in Figure 1.

The samples were distributed in two macro-clusters: A and B. Cluster A, the more heterogeneous, was divided in two sub-clusters, A1 and A2; A2 (orange) was formed of a single sample (Felina 32-2019), whilst A1 comprised two sub-groups, A1.1 and A1.2. The latter (evidenced in blue) was homogeneous, as it only included 2020 samples. Group A1.1, instead, resulted in two additional homogeneous groups (pink and green), of which the former mainly includes 2019 samples (except Felina 32-2020), while the latter only uses 2020 samples. Furthermore, cluster B (light blue) presented only one group, made up of 2019 samples, with the only exception of Uso-31.

The statistical distribution of the EOs samples evidenced a grouping based on the year of cultivation, rather than on the genotype, except for Uso-31, whose EOs of 2019 and 2020 occurred closely in the same cluster.

**Figure 2:**
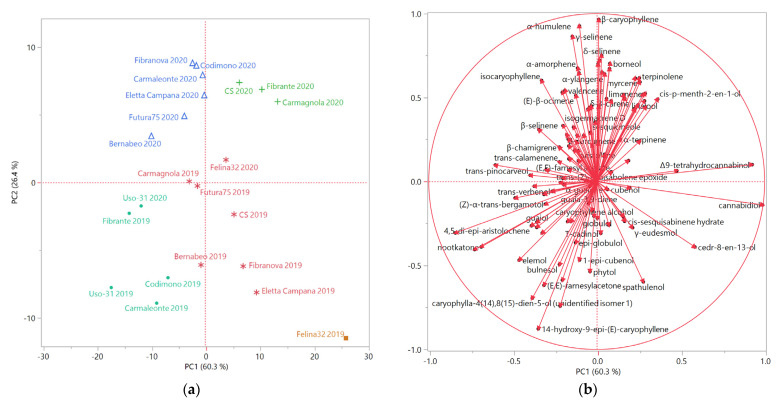
(**a**) Score plot of the principal component analysis (PCA) performed on the complete composition of the essential oils extracted from all the hemp samples; (**b**) Loading plot of the principal component analysis (PCA) performed on the complete composition of the essential oils extracted from all the hemp samples.

The score and loading plots obtained by the principal component analysis (PCA), performed on the complete chemical compositions of the EOs extracted from all the analyzed hemp samples, is reported in Figure 2a,b, respectively.

The distribution of the samples in the score plot evidenced a partition based on the year of cultivation, as the samples of 2019 were plotted in the bottom quadrants (PC2 < 0) of the score plot, and those of 2020 in the upper quadrants (PC2 > 0), with the exception of Uso-31-2020, which was placed in the bottom left quadrant (PC1 and PC2 < 0) as Uso-31-2019.

Furthermore, the grouping of the samples obtained by the PCA was comparable to that of the HCA. All the samples of the B macro-cluster, evidenced in light blue, were plotted in the bottom left quadrant (PC1 and PC2 < 0) of the PCA score plot, due to their relevant relative abundances in the oxygenated sesquiterpenes caryophyllene oxide, humulene oxide II, caryophylla-4(14),8(15)-dien-5-ol (isomer 1 or 2), and 14-hydroxy-9-*epi*-*(E)-*caryophyllene.

The samples of the A macro-cluster, instead, occupied the remaining three quadrants of the score plot, with few exceptions. In particular, the samples of the blue sub-cluster were plotted in the upper left quadrant (PC1 < 0 and PC2 > 0), while the EOs of the green cluster were positioned in the upper right quadrant (PC1 and PC2 > 0). The blue and the green sub-clusters included most of the 2020 EOs samples, excluding Uso-31-2020, as previously mentioned, and Felina 32-2020, belonging to the pink cluster, but also plotted in the upper right quadrant. The β-caryophyllene and α-humulene vectors were responsible for the positioning of the 2020 samples in the upper quadrants, whilst the discrimination between the left and the right quadrants was determined by minor sesquiterpeneshydrocarbons and monoterpenes vectors, respectively.

The only orange sub-cluster sample was plotted in the bottom right quadrant (PC1 > 0 and PC2 < 0), as three samples of the pink sub-group: this positioning was probably due to the cannabidiol vector. The other samples of the pink group were plotted very close to the bottom right quadrant: Bernabeo-2019 was plotted towards the rightmost area of the bottom left quadrant, due to its relevant relative abundances in cannabidiol, caryophyllene oxide and humulene oxide II. Although Bernabeo is reported as a CBG-chemotype cultivar, its presence in the EO was not detected: this was likely due to the lower volatility of CBG compared to CBD, its higher boiling point, and its overall higher polarity due to the positioning of its two hydroxyls on the benzenic ring, as they are less likely to engage in the formation of a pseudocyclic structure, as could, instead, occur in CBD. Finally, Futura 75-2019 and Carmagnola-2019, due to their relevant relative abundances in β-caryophyllene and α-humulene, were plotted next to each other, close to the PC1-PC2 axis intersection, on the left side of the score plot.

## 3. Materials and Methods

### 3.1. Plant Material

Plant material was obtained from experimental fields of CREA-CI located in Rovigo, Italy (45°04′43.4″ N 11°45′57.7″ E). For the two subsequent years of cultivation (2019–2020) the same fields were used, characterized by a cultural precession with straw cereals. Nitrogen fertilization was applied before sowing (40- and 54-units of nitrogen fertilizer in 2019 and 2020, respectively). Meteorological information (temperature and rainfall) was collected, and monthly averages are presented in Table 5.

The site had total rainfall during the outdoor growing season (from April to September) of 428 mm and 288 mm for 2019 and 2020, respectively. In the same period, the 2019 had an average temperature of 21.06 °C, slightly higher than those registered for the same period in 2020, when the average temperature 20.48 °C. In both years, the maximum temperature never exceeded 32 °C in the hottest months. At harvest, each plant was cut at its lower third and air-dried at ambient temperature and in the dark, to avoid photo-oxidation reactions. Material for EOs analysis included inflorescences and floral bracts, which were separated manually from stems and seeds, with a 2 mm diameter sieve.

### 3.2. Essential Oil Hydrodistillation

The extraction of the essential oil from the plant material was performed by hydrodistillation with a standard *Clevenger*-type apparatus, for 2 h. For both 2019 and 2020 samples, the hydrodistillation was carried out in triplicate, on 100 g of dried and shredded inflorescences and floral bracts, previously macerated in water under mechanical agitation (150 rpm) for 24 h prior to hydrodistillation. The collected essential oils were stored in a refrigerator until analysis.

### 3.3. Gas Chromatography–Mass Spectrometry Analyses and Peaks Identification

The essential oils were diluted to 0.5% in HPLC-grade *n*-hexane and then injected into a GC–MS apparatus. The GC–EIMS analyses were performed with an Agilent 7890B gas chromatograph (Agilent Technologies Inc., Santa Clara, CA, USA) equipped with an Agilent HP-5MS capillary column (30 m × 0.25 mm; coating thickness 0.25 µm) and an Agilent 5977B single quadrupole mass detector. The analytical conditions were as follows: oven temperature programmed from 60 to 240 °C at 3 °C/min; injector temperature, 220 °C; transfer line temperature, 240 °C; carrier gas helium, 1 mL/min. The injection volume was 1 μL, with a split ratio of 1:25. The acquisition parameters were as follows: full scan; scan range: 30–300 *m/z*; scan time: 1.0 s.

The identification of the constituents was based on a comparison between the retention times with those of the authentic samples, comparing their linear retention indices relative to the series of *n*-hydrocarbons. Computer matching was also used against commercial (NIST 14 and ADAMS 2007) and laboratory-developed mass spectra libraries built up from pure substances and components of commercial essential oils of known composition and the MS literature data [35,36,37,38,39,40].

### 3.4. Statistical Analyses

The percentage of dissimilarity contribution of all the compounds of *C. sativa* EOs was evaluated by means of the Similarity Percentage Test (SIMPER) with the Bray–Curtis distance/similarity measure. The statistical significance of the difference in the relative abundances of the compounds accounting for at least 1.00% in the dissimilarity rate of the emissions was evaluated using the F- or *t*-test, for compounds with equal or unequal variances, respectively. The SIMPER, F- and *t*-tests were performed with the Past 4.03 Software [41].

The analysis of variance (ANOVA) was carried out using the JMP Pro 14 software package (SAS Institute, Cary, NC, USA). A two-way ANOVA was carried out in order to estimate the variance components of genotypes (G; Bernabeo, Carmagnola, Carmaleonte, Codimono, CS, Eletta Campana, Felina 32, Fibranova, Fibrante, Futura 75, and Uso-31), year of cultivation (Y; 2019 and 2020), and their interaction (G × Y). Averages were separated by Tukey’s b *post-hoc* test. *p* < 0.05 was used to assess the significance of differences between means.

The multivariate statistical analyses were also carried out with the JMP software package. The covariance data matrix for the statistical evaluation of the EOs composition was a 116 × 22 matrix (116 compounds × 22 samples = 2.552 data). The principal component analysis (PCA) was performed selecting the two highest principal components (PCs) obtained by the linear regressions operated on mean-centered, unscaled data; as an unsupervised method, this analysis aimed at reducing the dimensionality of the multivariate data of the matrix, whilst preserving most of the variance. The principal component analysis (PCA) was performed, selecting the two highest PCs obtained by the linear regressions: the chosen PC1 and PC2 cover 60.30 and 26.40% of the variance, respectively, for a total explained variance of 86.70%. The hierarchical cluster analysis (HCA) was performed using Ward’s method, with squared Euclidean distances as a measure of similarity. Both the HCA and the PCA methods can be applied to observe groups of samples, even when there are no reference samples that can be used as a training set to establish the model.

## 4. Conclusions

The essential oils obtained from hemp inflorescences constituted a high-value derivative by-product, contributing to a more sustainable agricultural system. The present study aimed to evaluate the chemical composition and the yield of the EO obtained from eleven genotypes of hemp, cultivated and collected for two consecutive growing seasons in the same cultural conditions, to promote their employment based on their peculiar characteristics.

The results of the present study showed that sesquiterpenes and cannabinoids were the main classes of compounds of the hemp essential oils obtained from dried inflorescences. Regarding the sesquiterpenes, the hydrocarbon form was more abundant in 2020 samples, while the oxygenated one was predominant in 2019 EOs: the major compounds belonging to this chemical class were β-caryophyllene, α-humulene, and their oxygenated derivatives caryophyllene oxide, 14-hydroxy-9-*epi-(E)-*caryophyllene, and humulene oxide II, all typical components of hemp EO. The cannabinoids identified in all the samples were cannabidiol, cannabichromene, and Δ^9^-tetrahydrocannabinol, but only the former was revealed in relevant percentages. The EO extraction yield ranged from 0.03 to 0.12% *w/w* in 2019 samples, whilst in the 2020 ones, it was comprised between 0.03 and 0.23% *w/w*. The obtained data have shown that both the EO chemical profile and extraction yield were significantly influenced by the genotype of the starting material, the year of cultivation, and the interaction between these two factors.

## Figures and Tables

**Table 1:** Agronomic trial data from the years 2019–2020. Height is expressed in cm, the diameter at the collar in mm, the density as number of plants/m^2^, and the dried inflorescences yield is g/m^2^.

Genotype	Chemotype	Flower Type	Flowering Time	Sowing Date		Harvest Date		Height		Collar Diameter		Plant Density		Dried Inflorescences Yield	
				2019	2020	2019	2020	2019	2020	2019	2020	2019	2020	2019	2020
Carmaleonte	III	Monoecious	Medium	19 April	23 April	16 September	16 September	177 ± 42.5	223.6 ± 35.7	7.9 ± 2.8	13.8 ± 3.3	62 ± 24	2.2 ± 1.1	460	20
Codimono	III	Monoecious	Medium	19 April	23 April	25 September	16 September	183 ± 39.9	255.2 ± 32.8	6.6 ± 2.2	13.0 ± 3.6	77 ± 27.2	5.7 ± 0.7	440	51
Futura 75	III	Monoecious	Medium	19 April	23 April	18 September	16 September	77.3 ± 17.6	238.6 ± 58.2	255.6 ± 4.9	13.1 ± 4.4	8.6 ± 0.3	7.4 ± 1.3	71	67
Felina 32	III	Monoecious	Medium	4 June	23 April	20 September	16 September	151 ± 33	222.3 ± 43.3	5.9 ± 1.9	13.3 ± 4.5	61.3 ± 23.8	4.2 ± 1.5	160	40
Uso-31	V	Monoecious	Early	4 June	23 April	20 September	31 August	108 ± 31	184.4 ± 26.0	5.2 ± 1.9	11.6 ± 2.3	60.6 ± 11.3	3.2 ± 0.3	200	29
Bernabeo	IV	Dioecious	Late	19 April	23 April	28 September	8 October	211.6 ± 50.1	277.3 ± 38.9	8.6 ± 2.8	19.5 ± 6.7	64.3 ± 15.3	4.3 ± 3.2	430	39
Carmagnola	III	Dioecious	Late	19 April	23 April	9 October	8 October	228.3 ± 44.8	272.4 ± 34.5	8.2 ± 2.5	19.2 ± 5.1	61 ± 1	2.7 ± 2.0	410	24
Fibranova	III	Dioecious	Late	19 April	23 April	30 September	8 October	273.7 ± 45.6	314.5 ± 34.8	9.4 ± 2.8	17.1 ± 5.0	78.3 ± 14.4	6.3 ± 3.2	440	57
Fibrante	III	Dioecious	Late	19 April	23 April	11 October	8 October	269.1 ± 47.6	202.8 ± 5.2	10.1 ± 2.7	15.0 ± 1.3	85.3 ± 11	0.7 ± 0.4	420	6
Eletta Campana	III	Dioecious	Late	19 April	23 April	16 October	8 October	260.3 ± 44.3	304.5 ± 40.4	9.1 ± 2.6	17.5 ± 3.6	94.7 ± 29.1	5.7 ± 2.1	420	51
CS	III	Dioecious	Late	19 April	23 April	1 October	8 October	246 ± 51.8	333.7 ± 37.5	9.2 ± 3	18.1 ± 3.8	60.3 ± 22.3	6.3 ± 1.7	440	57

**Table 2:** Complete composition and extraction yield (% *w/w* dry weight) of the essential oil obtained from the dried inflorescences and floral bracts of 2019 hemp samples.

		Relative Abundance (%) ± SD										
		Monoecious					Dioecious					
Compounds	l.r.i. ^1^	Carmaleonte	Codimono	Felina 32	Futura 75	Uso-31	Bernabeo	Carmagnola	CS	Eletta Campana	Fibranova	Fibrante
heptanal	901	-	-	- ^2^	-	-	-	0.1 ± 0.06	-	-	-	0.1 ± 0.07
**α-pinene ^3^**	933	0.9 ± 0.21 ^AB;b^	0.5 ± 0.10 ^ABCD;b^	0.2 ± 0.00 ^BCD;b^	0.8 ± 0.30 ^AB;a^	0.8 ± 0.36 ^AB;b^	- ^D;b^	1.0 ± 0.46 ^A;b^	0.6 ± 0.09 ^ABCD;b^	0.2 ± 0.11 ^BCD;b^	0.1 ± 0.12 ^CD;b^	0.7 ± 0.10 ^ABC;b^
β-pinene	977	0.2 ± 0.05	0.1 ± 0.07	-	0.1 ± 0.05	0.2 ± 0.10	-	0.2 ± 0.09	0.1 ± 0.02	-	-	0.2 ± 0.01
**myrcene**	991	0.1 ± 0.01 ^BC;b^	0.1 ± 0.09 ^BC;b^	- ^C;b^	0.1 ± 0.07 ^BC;b^	0.2 ± 0.07 ^B;b^	- ^C;b^	0.5 ± 0.18 ^A;b^	0.3 ± 0.03 ^B;b^	- ^C;b^	- ^C;b^	0.6 ± 0.03 ^A;b^
δ-3-carene	1011	-	-	-	-	-	-	-	-	-	-	-
α-terpinene	1017	-	-	-	-	-	-	-	-	-	-	-
limonene	1029	-	-	-	-	0.1 ± 0.07	-	0.2 ± 0.01	0.2 ± 0.04	-	-	0.5 ± 0.01
1,8-cineole	1031	-	-	-	-	-	-	0.1 ± 0.01	0.1 ± 0.07	-	0.1 ± 0.06	-
*(E)*-β-ocimene	1047	-	-	-	-	0.1 ± 0.06	-	-	-	-	-	-
γ-terpinene	1058	-	-	-	-	-	-	-	0.1 ± 0.07	-	-	-
terpinolene	1089	-	-	-	-	-	-	-	0.1 ± 0.07	-	-	0.1 ± 0.01
nonanal	1105	-	-	-	-	0.1 ± 0.08	0.2 ± 0.04	0.1 ± 0.07	-	-	-	0.1 ± 0.06
linalool	1101	-	-	-	-	-	-	-	-	-	-	-
fenchol	1114	-	-	-	-	-	-	-	-	-	-	0.2 ± 0.04
*cis-p*-menth-2-en-1-ol	1122	-	-	-	-	-	-	-	-	-	-	-
*trans-*pinocarveol	1139	-	-	-	0.1 ± 0.05	0.2 ± 0.02	-	0.1 ± 0.09	-	-	-	0.1 ± 0.07
*trans-*verbenol	1145	-	-	-	-	-	-	-	-	-	-	-
ipsdienol	1147	-	-	-	-	-	-	-	-	-	-	-
β-pinene oxide	1156	-	-	-	-	-	-	-	-	-	-	0.1 ± 0.07
borneol	1165	-	-	-	-	-	-	0.1 ± 0.07	-	-	-	0.2 ± 0.05
lavandulol	1170	-	0.1 ± 0.07	0.1 ± 0.03	0.1 ± 0.08	-	-	-	0.2 ± 0.04	0.1 ± 0.07	0.1 ± 0.10	0.4 ± 0.10
4-terpineol	1177	-	-	-	-	-	-	0.1 ± 0.09	-	-	-	-
*p-*cymen-8-ol	1185	-	-	-	-	-	-	-	-	-	-	0.1±0.07
myrtenal	1194	-	-	-	-	0.1 ± 0.07	-	-	-	-	-	-
α-terpineol	1191	-	-	-	-	-	-	0.1 ± 0.09	0.3 ± 0.03	0.1 ± 0.08	0.1 ± 0.08	0.3 ± 0.09
eugenol	1357	0.2 ± 0.04	-	-	0.1 ± 0.05	-	-	-	-	-	0.2 ± 0.20	-
methyl eugenol	1405	-	-	-	-	-	-	-	-	-	-	-
α-ylangene	1371	-	-	-	-	-	-	0.1 ± 0.05	-	-	-	-
*iso*caryophyllene	1407	0.2 ± 0.01	0.3 ± 0.01	0.1 ± 0.01	0.4 ± 0.02	0.2 ± 0.08	-	0.4 ± 0.07	0.3 ± 0.01	0.2 ± 0.04	0.2 ± 0.06	0.5 ± 0.11
*cis*-α-bergamotene	1416	-	-	-	0.1 ± 0.06	-	-	-	-	-	-	-
**β-caryophyllene**	1419	4.6 ± 0.21 ^C;b^	6.6 ± 0.17 ^BC;b^	2.9 ± 0.11 ^C;b^	11.0 ± 0.70 ^A;b^	4.9 ± 1.29 ^C;b^	3.6 ± 0.62 ^C;b^	10.2 ± 2.72 ^AB;a^	9.4 ± 1.66 ^AB;b^	3.1 ± 0.54 ^C;b^	6.3 ± 2.32 ^BC;b^	10.2 ± 1.27 ^AB;b^
*trans*-α-bergamotene	1436	-	0.5 ± 0.01	0.3 ± 0.02	1 ± 0.02	0.5 ± 0.16	-	0.1 ± 0.07	-	0.1 ± 0.07	0.4 ± 0.17	-
α-guaiene	1439	-	-	-	-	-	0.3 ± 0.09	0.1 ± 0.07	-	-	0.1 ± 0.08	-
aromadendrene	1142	0.1 ± 0.01	-	-	-	-	-	0.1 ± 0.05	-	-	-	-
guaia-6,9-diene	1443	-	-	-	-	-	-	-	-	-	-	-
*iso*germacrene D	1451	-	-	-	-	-	-	-	-	-	-	-
**α-humulene**	1453	2.6 ± 0.08 ^DE;b^	3.7 ± 0.10 ^BCD;b^	1.7 ± 0.12 ^E;b^	5.0 ± 0.12 ^AB;b^	2.9 ± 0.73 ^CDE;b^	2.7 ± 0.46 ^CDE;b^	4.3 ± 0.99 ^ABC;a^	5.0 ± 0.50 ^AB;b^	1.8 ± 0.42 ^E;b^	2.9 ± 1.04 ^CDE;b^	5.7 ± 0.74 ^A;b^
aristolene	1452	-	-	-	-	-	-	0.1 ± 0.07	-	-	-	-
*(E)*-β-farnesene	1458	-	0.3 ± 0.01	0.3 ± 0.03	0.8 ± 0.11	0.5 ± 0.18	-	0.1 ± 0.06	-	-	0.3 ± 0.08	-
*allo*aromadendrene	1460	0.2 ± 0.02	0.7 ± 0.03	-	0.8 ± 0.06	1.4 ± 0.44	0.2 ± 0.04	0.6 ± 0.15	0.4 ± 0.02	0.3 ± 0.04	0.3 ± 0.09	0.3 ± 0.05
4,5-di-*epi*-aristolochene	1468	-	-	-	-	0.1 ± 0.06	-	-	-	-	-	-
γ-gurjunene	1469	-	-	-	0.1 ± 0.07	-	-	-	-	-	-	-
β-chamigrene	1476	-	-	-	-	-	-	-	-	-	-	-
γ-muurolene	1477	0.4 ± 0.06	0.2 ± 0.01	0.1 ± 0.01	0.2 ± 0.03	0.1 ± 0.10	-	0.2 ± 0.06	0.1 ± 0.08	0.4 ± 0.16	0.2 ± 0.08	-
α-amorphene	1482	-	0.1 ± 0.06	-	0.1 ± 0.01	-	-	-	-	-	-	-
γ-selinene	1483	0.1 ± 0.01	0.3 ± 0.02	0.2 ± 0.03	0.5 ± 0.01	0.3 ± 0.08	0.3 ± 0.05	0.3 ± 0.13	0.3 ± 0.04	0.2 ± 0.05	0.2 ± 0.09	0.3 ± 0.03
**β-selinene**	1486	0.8 ± 0.03 ^B;b^	0.9 ± 0.02 ^B;b^	0.9 ± 0.11 ^B;b^	2.2 ± 0.05 ^A;a^	2.4 ± 0.65 ^A;a^	0.9 ± 0.13 ^B;b^	1.2 ± 0.38 ^B;a^	1.1 ± 0.07 ^B;b^	1.2 ± 0.40 ^B;a^	0.9 ± 0.36^B;a^	1.0 ± 0.14 ^B;b^
δ-selinene	1491	-	-	-	-	-	-	-	-	-	-	-
valencene	1493	0.2 ± 0.03	0.2 ± 0.07	0.1 ± 0.05	0.2 ± 0.05	0.2 ± 0.01	0.1 ± 0.09	-	0.4 ± 0.02	0.1 ± 0.02	0.1 ± 0.08	0.3 ± 0.06
α-selinene	1495	0.4 ± 0.01	0.5 ± 0.03	0.5 ± 0.07	1.5 ± 0.09	1.1 ± 0.32	0.6 ± 0.13	0.7 ± 0.11	0.7 ± 0.09	0.8 ± 0.23	0.6 ± 0.27	0.6 ± 0.06
eremophilene	1499	-	-	-	-	-	-	-	-	-	-	-
α-bulnesene	1505	-	-	-	-	-	0.6 ± 0.06	0.1 ± 0.09	-	0.1 ± 0.07	0.1 ± 0.10	-
*epi*zonarene	1501	-	-	-	-	-	-	-	-	-	-	-
β-bisabolene	1509	-	0.1 ± 0.02	-	0.2 ± 0.02	0.1 ± 0.06	-	0.3 ± 0.08	0.1 ± 0.02	0.2 ± 0.06	0.2 ± 0.08	0.2 ± 0.03
*(E,E)*-α-farnesene	1509	-	-	-	-	-	-	-	-	-	-	-
β-curcumene	1513	-	-	-	0.2 ± 0.03	-	-	-	-	-	-	-
*trans*-γ-cadinene	1513	0.3 ± 0.01	0.2 ± 0.05	0.1 ± 0.09	0.3 ± 0.04	0.3 ± 0.07	-	0.2 ± 0.03	0.1 ± 0.06	0.3 ± 0.10	0.1 ± 0.02	0.1 ± 0.02
sesquicineole	1516	-	-	-	-	-	-	-	-	-	-	-
7-*epi-*α-selinene	1517	0.2 ± 0.03	0.2 ± 0.04	-	0.3 ± 0.03	0.1 ± 0.15	-	0.4 ± 0.09	0.3 ± 0.01	0.1 ± 0.12	-	0.3 ± 0.04
*(Z)-*γ-bisabolene	1519	-	-	-	-	-	-	-	-	-	0.1 ± 0.11	-
β-cadinene	1519	-	-	-	0.3 ± 0.04	-	-	0.1 ± 0.03	0.1 ± 0.07	0.1 ± 0.12	0.2 ± 0.01	0.1 ± 0.07
*trans*-calamenene	1524	-	-	-	-	-	-	-	-	-	-	-
δ-cadinene	1524	0.2 ± 0.02	0.2 ± 0.04	0.1 ± 0.06	0.5 ± 0.02	0.1 ± 0.08	-	0.2 ± 0.06	0.2 ± 0.01	0.3 ± 0.08	0.2 ± 0.01	0.1 ± 0.10
**selina-3,7(11)-diene**	1530	1.6 ± 0.10 ^ABCD;b^	1.0 ± 0.11 ^BCD;b^	0.6 ± 0.14 ^D;b^	2.1 ± 0.59 ^A;a^	0.9 ± 0.16 ^CD;b^	1.6 ± 0.31 ^ABCD;b^	2.5 ± 0.21 ^AB;b^	2.1 ± 0.06 ^ABCD;b^	2.3 ± 0.33 ^ABC;b^	2.1 ± 0.59 ^ABC;b^	1.9 ± 0.16 ^ABCD;a^
*(E)*-γ-bisabolene	1531	-	0.5 ± 0.05	-	-	-	-	-	-	-	-	-
α-calacorene	1543	0.2 ± 0.01	-	-	0.1 ± 0.14	-	0.2 ± 0.03	0.4 ± 0.06	0.3 ± 0.07	0.2 ± 0.01	-	0.3 ± 0.04
*cis*-sesquisabinene hydrate	1545	-	-	-	0.1 ± 0.02	-	-	-	-	0.1 ± 0.08	0.6 ± 0.18	-
elemol	1550	0.8 ± 0.13	0.7 ± 0.07	0.4 ± 0.06	1.1 ± 0.14	1.0 ± 0.24	0.2 ± 0.05	0.7 ± 0.00	0.5 ± 0.05	0.3 ± 0.06	0.4 ± 0.08	0.6 ± 0.03
guaia-3,9-diene	1556	-	0.1 ± 0.01	-	-	-	-	-	-	-	-	-
*(E)*-nerolidol	1564	0.7 ± 0.05	0.7 ± 0.05	0.5 ± 0.16	1.2 ± 0.31	0.6 ± 0.14	1.3 ± 0.28	0.6 ± 0.07	1.0 ± 0.07	1.0 ± 0.25	1.5 ± 0.30	1.1 ± 0.13
palustrol	1568	-	-	-	-	-	-	-	-	-	-	-
caryophyllene alcohol	1570	-	-	-	-	-	0.9 ± 0.12	-	-	-	-	-
spathulenol	1577	0.1 ± 0.00	0.2 ± 0.03	0.2 ± 0.07	-	-	-	-	-	-	0.1 ± 0.00	-
*trans*-sesquisabinene hydrate	1581	-	-	-	0.1 ± 0.07	-	-	-	-	-	-	-
**caryophyllene oxide**	1582	16.0 ± 0.1 ^B;a^	14.2 ± 0.82 ^BC;a^	7.1 ± 1.39 ^D;a^	10.2 ± 1.32 ^CD;b^	23.5 ± 3.19 ^A;a^	14.2 ± 1.83 ^BC;a^	13.7 ± 0.17 ^BC;a^	11.3 ± 0.85 ^CD;a^	10.9 ± 2.56 ^CD;a^	10.2 ± 1.12 ^CD;a^	16.6 ± 0.78 ^B;a^
globulol	1583	-	-	-	-	-	0.6 ± 0.09	-	0.1 ± 0.06	-	-	-
*trans-(Z)*-α-bisabolene epoxide	1586	-	-	-	-	-	-	-	-	-	-	-
*iso*aromadendrene epoxide	1589	-	-	-	0.9 ± 0.23	-	-	-	0.9 ± 0.13	-	-	0.6 ± 0.60
*epi-*globulol	1590	-	-	0.2 ± 0.04	-	0.2 ± 0.03	-	0.5 ± 0.32	-	0.6 ± 0.01	0.2 ± 0.01	0.9 ± 0.91
viridiflorol	1592	0.3 ± 0.03	0.5 ± 0.07	-	0.5 ± 0.11	-	-	0.6 ± 0.20	0.3 ± 0.05	2.7 ± 2.20	0.3 ± 0.01	0.1 ± 0.10
guaiol	1596	-	-	-	-	1.1 ± 0.10	-	-	-	-	-	-
**humulene oxide II**	1608	6.1 ± 0.47 ^BC;a^	6.6 ± 0.52 ^AB;a^	3.7 ± 0.94 ^CD;a^	4.8 ± 0.41 ^BCD;a^	9.1 ± 0.76 ^A;a^	6.9 ± 0.73 ^AB;a^	2.7 ± 2.40 ^D;a^	4.5 ± 0.56 ^BCD;a^	2.6 ± 1.07 ^D;a^	4.2 ± 0.68 ^BCD;a^	7.0 ± 0.07 ^AB;a^
cedrenol	1610	-	-	-	-	-	1.0 ± 0.04	-	-	-	-	0.2 ± 0.20
humulane-1-6-dien-3-ol	1613	-	-	-	-	0.1 ± 0.09	-	-	-	-	-	-
**selin-6-en-4-ol**	1618	2.4 ± 1.09 ^AB;a^	1.6 ± 0.56 ^ABCD;a^	0.4 ± 0.05 ^D;a^	1.5 ± 0.25 ^BCD;a^	0.8 ± 0.11 ^CD;a^	3.1 ± 0.77 ^A;a^	1.7 ± 0.33 ^ABCD;a^	1.3 ± 0.13 ^BCD;a^	1.8 ± 0.06 ^ABCD;a^	1.7 ± 0.28 ^ABCD;a^	2.3 ± 0.70 ^ABC;a^
13-*nor*-valenc-1(10)-en-11-one	1629	-	-	-	-	-	-	-	-	-	-	-
1-*epi*-cubenol	1627	1.0 ± 0.12	-	0.4 ± 0.12	1.0 ± 0.37	0.6 ± 0.00	0.8 ± 0.23	1.0 ± 0.78	0.2 ± 0.18	1.0 ± 0.49	0.6 ± 0.07	0.2 ± 0.25
γ-eudesmol	1631	-	-	-	-	-	-	-	-	0.3 ± 0.31	-	-
**caryophylla-4(14),8(15)-dien-5-ol (unidentified isomer 1)**	1633	8.3 ± 0.12 ^A;a^	7.6 ± 0.37 ^AB;a^	3.8 ± 0.78 ^CD;a^	4.9 ± 1.83 ^ABCD;a^	4.7 ± 0.35 ^BCD;a^	2.5 ± 0.39 ^D;a^	3.3 ± 2.85 ^D;a^	3.8 ± 0.65 ^CD;a^	3.5 ± 0.81 ^D;a^	5.0 ± 0.36 ^ABCD;a^	7.1 ± 0.90 ^ABC;a^
**caryophylla-4(14),8(15)-dien-5-ol (unidentified isomer 2)**	1633	7.3 ± 0.18 ^A;a^	5.7 ± 0.51 ^B;a^	3.9 ± 0.65 ^DE;a^	3.7 ± 0.57 ^E;a^	5.4 ± 0.27 ^BC;a^	- ^F;b^	4.1 ± 0.68 ^CDE;a^	4.1 ± 0.35 ^CDE;a^	3.5 ± 0.13 ^E;a^	4.8 ± 0.24 ^BCDE;a^	5.1 ± 0.65 ^BCD;a^
T-cadinol	1641	-	-	-	-	0.2 ± 0.03	0.1 ± 0.11	-	-	0.3 ± 0.12	-	-
cubenol	1641	0.1 ± 0.11	-	-	-	-	-	0.1 ± 0.07	-	-	0.4 ± 0.02	-
β-eudesmol	1649	0.2 ± 0.05	0.1 ± 0.08	-	-	0.1 ± 0.06	0.2 ± 0.21	0.7 ± 0.59	0.1 ± 0.06	-	-	-
α-eudesmol	1653	-	-	-	-	-	-	0.3 ± 0.19	-	-	-	-
*neo*intermedeol	1655	0.2 ± 0.01	-	0.2 ± 0.22	-	0.5 ± 0.00	1.7 ± 0.11	-	-	-	0.5 ± 0.01	-
bulnesol	1668	0.4 ± 0.01	0.3 ± 0.07	-	-	-	-	-	-	0.2 ± 0.24	-	0.1 ± 0.11
**14-hydroxy-9-*epi-(E)*-caryophyllene**	1670	13.5 ± 0.42 ^A;a^	13.5 ± 0.71 ^A;a^	9.2 ± 2.34 ^BCD;a^	9.1 ± 0.46 ^BCD;a^	12.2 ± 1.22 ^ABC;a^	8.1 ± 0.67 ^CD;a^	7.8 ± 2.73 ^D;a^	8.7 ± 1.99 ^BCD;a^	9.1 ± 1.33 ^BCD;a^	10.2 ± 0.44 ^ABCD;a^	12.6 ± 0.14 ^AB;a^
ylangenal	1675	-	0.1 ± 0.08	-	-	0.2 ± 0.01	-	0.1 ± 0.08	-	0.1 ± 0.01	0.2 ± 0.03	-
aromadendrene epoxide II	1680	0.6 ± 0.01	0.6 ± 0.04	0.3 ± 0.11	0.3 ± 0.03	0.5 ± 0.1	0.3 ± 0.04	0.3 ± 0.11	0.4 ± 0.07	0.4 ± 0.07	0.3 ± 0.01	0.4 ± 0.05
**α-bisabolol**	1685	0.5 ± 0.02 ^FG;a^	2.6 ± 0.20 ^C;a^	0.4 ± 0.20 ^FG;b^	0.8 ± 0.17 ^EF;a^	- ^G;b^	1.2 ± 0.22 ^DE;a^	1.6 ± 0.38 ^D;a^	1.3 ± 0.13 ^DE;a^	4.9 ± 0.30 ^A;a^	4.0 ± 0.29 ^B;a^	0.8 ± 0.06 ^EF;b^
cedr-8-en-13-ol	1688	-	-	0.1 ± 0.08	-	-	-	-	-	-	-	-
juniper camphor	1694	1.0 ± 0.02	0.9 ± 0.02	0.2 ± 0.07	0.6 ± 0.08	0.2 ± 0.02	1.2 ± 0.11	0.6 ± 0.11	1.0 ± 0.31	1.1 ± 0.30	0.9 ± 0.05	0.9 ± 0.03
(Z)-α-*trans*-bergamotol	1700	0.2 ± 0.01	-	-	-	-	-	-	-	-	-	-
α-phellandrene dimer	1801	-	-	-	-	-	-	-	-	-	-	-
nootkatone	1803	0.2 ± 0.00	0.2 ± 0.01	-	-	0.2 ± 0.04	-	-	-	-	-	0.1 ± 0.06
*(E,E)*-farnesyl acetate	1843	-	-	-	-	-	-	-	-	-	-	-
**hexahydrofarnesylacetone**	1845	0.7 ± 0.05 ^C;a^	1.4 ± 0.05 ^BC;a^	1.0 ± 0.29 ^C;b^	1.0 ± 0.03 ^C;a^	1.9 ± 0.72 ^AB;a^	2.3 ± 0.04 ^A;a^	2.3 ± 0.02 ^A;a^	2.0 ± 0.09 ^AB;a^	1.4 ± 0.34 ^BC;a^	0.7 ± 0.07 ^C;a^	1.4 ± 0.21 ^BC;a^
1-hexadecanol	1877	-	-	-	-	-	-	-	-	-	-	-
*(E,E)*-farnesyl acetone	1918	0.4 ± 0.03	0.3 ± 0.00	0.7 ± 0.22	0.4 ± 0.03	1.6 ± 0.04	0.5 ± 0.02	0.4 ± 0.01	0.3 ± 0.04	0.2 ± 0.07	0.1 ± 0.00	0.3 ± 0.05
*m-*camphorene	1952	-	-	-	-	-	-	0.3 ± 0.02	0.1 ± 0.06	-	-	0.1 ± 0.07
*p-*camphorene	1986	-	-	-	-	-	-	0.6 ± 0.06	-	-	-	0.1 ± 0.12
ethyl hexadecanoate	2000	-	-	-	-	-	-	-	-	-	-	-
**phytol**	2112	0.9 ± 0.10 ^DE;a^	1.5 ± 0.03 ^CDE;a^	3.1 ± 0.65 ^B;a^	0.6 ± 0.03 ^E;a^	2.8 ± 0.18 ^B;b^	5.4 ± 0.91 ^A;a^	1.1 ± 0.30 ^CDE;a^	2.1 ± 0.45 ^BC;a^	2.0 ± 0.15 ^BCD;a^	0.8 ± 0.09 ^E;a^	0.9 ± 0.25 ^E;a^
**cannabidiol**	2369	18.1 ± 1.7 ^BC;a^	19.6 ± 1.23 ^BC;a^	51.5 ± 8.36 ^A;a^	22.9 ± 6.33 ^BC;a^	11.8 ± 7.97 ^C;a^	25.0 ± 5.7 ^BC;a^	21.9 ± 0.92 ^BC;a^	30.5 ± 1.62 ^B;a^	34.5 ± 7.88 ^B;a^	32.1 ± 9.62 ^B;a^	12.6 ± 1.07 ^C;b^
cannabichromene	2373	0.5 ± 0.18	0.2 ± 0.07	1.2 ± 0.96	0.3 ± 0.18	0.7 ± 0.56	1.6 ± 0.26	0.3 ± 0.18	0.5 ± 0.40	0.2 ± 0.01	1.0 ± 0.43	0.2 ± 0.16
Δ^9^-tetrahydrocannabinol	2468	0.2 ± 0.06	0.3 ± 0.09	0.8 ± 0.15	0.3 ± 0.10	0.1 ± 0.13	0.3 ± 0.07	0.3 ± 0.05	0.4 ± 0.06	0.4 ± 0.10	0.3 ± 0.13	0.1 ± 0.01
Total identified (%)		93.8 ± 0.43	96.4 ± 0.12	97.4 ± 0.98	95.9 ± 1.42	97.3 ± 0.24	90.6 ± 0.92	92.5 ± 1.13	97.8 ± 0.52	95.4 ± 1.95	97.1 ± 0.79	97.7 ± 0.65
Monoterpene hydrocarbons		1.2 ± 0.26 ^ABC;b^	0.7 ± 0.25 ^BCD;b^	0.2 ± 0.00 ^CD;b^	0.9 ± 0.42 ^BCD;b^	1.4 ± 0.65 ^AB;b^	^-D;b^	2.0 ± 0.73 ^A;b^	1.3 ± 0.31 ^AB;b^	0.2 ± 0.11 ^CD;b^	0.1 ± 0.12 ^D;b^	2.1 ± 0.12 ^A;b^
Oxygenated monoterpenes		^-B;b^	0.1 ± 0.07 ^B;a^	0.1 ± 0.03 ^B;b^	0.1 ± 0.13 ^B;a^	0.2 ± 0.09 ^B;b^	^-B;b^	0.5 ± 0.32 ^B;b^	0.6 ± 0.13 ^B;b^	0.1 ± 0.14 ^B;b^	0.2 ± 0.24 ^B;b^	1.3 ± 0.47 ^A;a^
Sesquiterpene hydrocarbons		12.1 ± 0.23 ^CD;b^	16.4 ± 0.66 ^BCD;b^	8.0 ± 0.34 ^D;b^	28.7 ± 0.84 ^A;b^	15.9 ± 4.57 ^BCD;a^	11.0 ± 1.99 ^D;b^	22.3 ± 5.37 ^AB;a^	20.8 ± 2.42 ^ABC;b^	11.7 ± 2.83 ^D;b^	15.5 ± 5.37 ^BCD;b^	21.8 ± 2.90 ^AB;b^
Oxygenated sesquiterpenes		59.6 ± 1.79 ^A;a^	56.0 ± 1.50 ^AB;a^	30.9 ± 7.02 ^D;a^	40.6 ± 4.82 ^CD;a^	60.9 ± 2.48 ^A;a^	44.3 ± 3.81 ^BCD;a^	40.5 ± 8.52 ^CD;a^	39.3 ± 3.68 ^CD;a^	44.6 ± 6.31 ^BC;a^	46.0 ± 3.49 ^BC;a^	56.7 ± 1.86 ^AB;a^
Diterpene hydrocarbons		- ^C;b^	- ^C;a^	- ^C;b^	- ^C;b^	- ^C^	^-C;b^	0.9 ± 0.08 ^A;a^	0.1 ± 0.06 ^BC;b^	- ^C;b^	- ^C^	0.2 ± 0.19 ^B;b^
Oxygenated diterpenes		0.9 ± 0.10 ^DE;a^	1.5 ± 0.03 ^CDE;a^	3.1 ± 0.65 ^B;a^	0.6 ± 0.03 ^E;a^	2.8 ± 0.18 ^B;b^	5.4 ± 0.91 ^A;a^	1.1 ± 0.30 ^CDE;a^	2.1 ± 0.45 ^BC;a^	2.0 ± 0.15 ^BCD;a^	0.8 ± 0.09 ^E;a^	0.9 ± 0.25 ^E;a^
Phenylpropanoids		0.2 ± 0.04 ^A;a^	- ^B;a^	- ^B^	0.1 ± 0.05 ^AB;a^	- ^B,b^	^B^	- ^B^	- ^B^	- ^B^	0.2 ± 0.20 ^A;a^	- ^B^
Cannabinoids		18.7 ± 1.93 ^BC;a^	20.0 ± 1.24 ^BC;a^	53.4 ± 9.47 ^A;a^	23.5 ± 6.61 ^BC;a^	12.6 ± 8.66 ^C;a^	26.9 ± 5.89 ^BC;a^	22.5 ± 1.05 ^BC;b^	31.4 ± 1.97 ^B;a^	35.1 ± 7.99 ^B;a^	33.5 ± 10.18 ^B;a^	12.9 ± 1.22 ^C;b^
Apocarotenoids		1.1 ± 0.07 ^D;a^	1.7 ± 0.05 ^CD;a^	1.7 ± 0.51 ^CD;a^	1.4 ± 0.01 ^CD;a^	3.5 ± 0.76 ^A;a^	2.8 ± 0.06 ^AB;a^	2.8 ± 0.01 ^AB;a^	2.2 ± 0.13 ^BC;a^	1.6 ± 0.41 ^CD;a^	0.8 ± 0.07 ^D;a^	1.7 ± 0.26 ^CD;a^
Other non-terpene derivatives		- ^A;a^	- ^A^	- ^A;a^	- ^A^	0.1 ± 0.08 ^A;a^	0.2 ± 0.04 ^A;b^	0.1 ± 0.15 ^A;a^	- ^A;b^	- ^A;a^	- ^A^	0.1 ± 0.13 ^A;a^
Extraction yield (% *w/w*)		0.04 ± 0.02 ^DE;b^	0.07 ± 0.01 ^BCD;b^	0.03 ± 0.01 ^E;b^	0.12 ± 0.01 ^A;b^	0.03 ± 0.00 ^E^	0.03 ± 0.01 ^E;b^	0.09 ± 0.02 ^AB;b^	0.08 ± 0.01 ^BC;b^	0.09 ± 0.00 ^AB^	0.06 ± 0.01 ^CDE;b^	0.08 ± 0.00 ^BC;b^

^1^ Linear retention index on a HP 5-MS capillary column; ^2^ Not detected; ^3^ Compounds accounting for at least 1.000% of the dissimilarity rate (According to the SIMPER test, see Table 4) are evidenced in bold. For these compounds, for all chemical classes, and for the extraction yield, different superscript uppercase letters (A–G) indicate statistically significant differences between the each variety; superscript lowercase letters (a,b) indicate statistically significant differences among the cultivar withdrawn on different years (see Table 3 for 2020 samples). The statistical significance of the relative abundances was established by the Tukey’s *post-hoc* test, with *p* ≤ 0.05.

**Table 3:** Complete composition and extraction yield (% *w/w* dry weight) of the essential oil obtained from the dried inflorescences and floral bracts of 2020 hemp samples.

		Relative Abundance (%) ± SD										
		Monoecious					Dioecious					
Compounds	l.r.i. ^1^	Carmaleonte	Codimono	Felina32	Futura 75	Uso-31	Bernabeo	Carmagnola	CS	Eletta Campana	Fibranova	Fibrante
heptanal	901	- ^2^	-	-	-	-	0.3 ± 0.06	0.1 ± 0.10	0.1 ± 0.04	0.1 ± 0.07	-	-
**α-pinene ^3^**	933	2.7 ± 0.50 ^AB;a^	2.0 ± 0.74 ^ABC;a^	1.1 ± 0.40 ^CD;a^	1.1 ± 0.11 ^CD;a^	2.0 ± 0.26 ^ABC;a^	0.5 ± 0.06 ^D;a^	3.0 ± 0.04 ^A;a^	2.1 ± 0.79 ^ABC;a^	1.7 ± 0.01 ^BC;a^	1.3 ± 0.39 ^CD;a^	1.2 ± 0.06 ^CD;a^
β-pinene	977	0.5 ± 0.06	0.5 ± 0.17	0.3 ± 0.07	0.2 ± 0.06	0.7 ± 0.18	0.1 ± 0.01	0.8 ± 0.09	0.7 ± 0.26	0.5 ± 0.02	0.3 ± 0.12	0.4 ± 0.03
**myrcene**	991	0.8 ± 0.09 ^CD;a^	0.8 ± 0.33 ^CD;a^	0.2 ± 0.02 ^D;a^	0.4 ± 0.09 ^D;a^	0.6 ± 0.12 ^D;a^	0.4 ± 0.05 ^D;a^	2.6 ± 0.16 ^A;a^	1.3 ± 0.49 ^BC;a^	0.6 ± 0.01 ^D;a^	0.3 ± 0.10 ^D;a^	1.8 ± 0.17 ^B;a^
δ-3-carene	1011	0.1 ± 0.05	-	-	-	-	-	-	-	0.1 ± 0.01	0.3 ± 0.09	-
α-terpinene	1017	-	-	-	-	-	-	-	0.1 ± 0.06	-	-	-
limonene	1029	0.1 ± 0.01	0.1 ± 0.08	-	-	0.2 ± 0.01	0.1 ± 0.03	0.6 ± 0.10	0.7 ± 0.16	0.4 ± 0.01	0.1 ± 0.08	0.8 ± 0.08
1,8-cineole	1031	-	0.2 ± 0.05	-	-	-	0.2 ± 0.05	0.2 ± 0.09	0.2 ± 0.06	0.1 ± 0.02	0.2 ± 0.06	0.1 ± 0.06
*(E)*-β-ocimene	1047	0.1 ± 0.01	0.4 ± 0.13	-	0.1 ± 0.03	0.4 ± 0.02	-	0.3 ± 0.02	0.1 ± 0.06	0.2 ± 0.01	0.3 ± 0.07	-
γ-terpinene	1058	-	-	-	-	-	-	-	0.1 ± 0.07	-	-	-
terpinolene	1089	-	0.4 ± 0.14	0.2 ± 0.04	0.1 ± 0.09	0.1 ± 0.02	0.1 ± 0.01	0.3 ± 0.03	0.8 ± 0.19	0.3 ± 0.01	-	0.5 ± 0.03
nonanal	1105	0.1 ± 0.07	-	-	-	0.1 ± 0.06	0.2 ± 0.04	-	-	-	-	-
linalool	1101	-	0.1 ± 0.06	-	-	-	-	0.2 ± 0.01	0.2 ± 0.04	0.1 ± 0.03	-	-
fenchol	1114	-	-	-	-	-	-	0.2 ± 0.02	0.4 ± 0.07	0.2 ± 0.05	-	0.3 ± 0.00
*cis-p*-menth-2-en-1-ol	1122	-	-	-	-	-	-	0.2 ± 0.02	0.2 ± 0.03	0.2 ± 0.02	-	0.2 ± 0.01
*trans-*pinocarveol	1139	0.3 ± 0.03	-	-	-	0.1 ± 0.01	-	-	-	-	-	-
*trans-*verbenol	1145	-	-	-	-	0.2 ± 0.01	-	-	-	-	-	-
ipsdienol	1147	0.1 ± 0.05	0.1 ± 0.07	-	-	-	-	-	0.1 ± 0.07	-	-	-
β-pinene oxide	1156	-	-	-	-	-	-	0.3 ± 0.03	-	-	-	0.1 ± 0.01
borneol	1165	0.2 ± 0.02	0.1 ± 0.05	-	-	-	0.1 ± 0.00	0.2 ± 0.02	0.2 ± 0.04	0.2 ± 0.00	0.1 ± 0.06	0.2 ± 0.01
lavandulol	1170	-	0.4 ± 0.09	0.3 ± 0.06	0.2 ± 0.05	0.2 ± 0.05	0.4 ± 0.06	0.3 ± 0.01	0.6 ± 0.03	0.3 ± 0.01	-	0.6 ± 0.04
4-terpineol	1177	-	-	-	-	-	-	-	-	-	0.2 ± 0.03	-
*p-*cymen-8-ol	1185	-	-	-	-	-	-	-	-	-	-	-
myrtenal	1194	-	-	-	-	-	-	-	-	-	-	-
α-terpineol	1191	-	0.2 ± 0.03	-	-	-	0.2 ± 0.04	0.3 ± 0.01	0.5 ± 0.04	-	0.2 ± 0.03	0.5 ± 0.02
eugenol	1357	0.2 ± 0.06	0.1 ± 0.07	-	0.1 ± 0.03	0.1 ± 0.01	-	-	-	-	0.2 ± 0.07	-
methyl eugenol	1405	-	-	-	-	0.1 ± 0.02	-	-	-	-	-	-
α-ylangene	1371	0.2 ± 0.01	0.1 ± 0.07	-	0.1 ± 0.01	-	-	0.1 ± 0.04	0.1 ± 0.02	0.3 ± 0.01	0.1 ± 0.07	-
*iso*caryophyllene	1407	0.4 ± 0.03	0.4 ± 0.00	0.3 ± 0.09	0.4 ± 0.03	0.2 ± 0.01	0.3 ± 0.11	0.2 ± 0.04	0.3 ± 0.06	0.3 ± 0.01	0.5 ± 0.12	0.3 ± 0.01
*cis*-α-bergamotene	1416	-	0.1 ± 0.01	0.4 ± 0.14	0.1 ± 0.09	-	-	-	-	-	-	-
**β-caryophyllene**	1419	15.3 ± 1.44 ^AB;a^	16.2 ± 1.46 ^AB;a^	11.5 ± 3.55 ^ABC;a^	13.8 ± 0.59 ^ABC;a^	8.7 ± 0.77 ^C;a^	10.4 ± 2.95 ^BC;a^	14.4 ± 0.89 ^ABC;a^	15.4 ± 2.45 ^AB;a^	11.6 ± 0.09 ^ABC;a^	16.8 ± 3.82 ^A;a^	16.2 ± 0.8 ^AB;a^
*trans*-α-bergamotene	1436	0.3 ± 0.00	1.1 ± 0.04	1.6 ± 0.42	1.5 ± 0.16	1.0 ± 0.06	0.3 ± 0.06	0.1 ± 0.01	0.1 ± 0.08	0.3 ± 0.01	0.9 ± 0.2	-
α-guaiene	1439	-	-	-	-	-	0.6 ± 0.14	-	-	-	-	-
aromadendrene	1142	-	-	-	-	-	-	-	-	-	0.3 ± 0.04	-
guaia-6,9-diene	1443	0.2 ± 0.01	-	-	-	-	-	-	-	0.1 ± 0.05	0.1 ± 0.05	-
*iso*germacrene D	1451	0.3 ± 0.03	-	-	-	-	-	-	-	0.2 ± 0.01	-	-
**α-humulene**	1453	6.9 ± 1.00 ^ABC;a^	7.6 ± 0.17 ^A;a^	4.7 ± 1.23 ^DE;a^	5.8 ± 0.38 ^ABCDE;a^	4.1 ± 0.06 ^E;a^	6.2 ± 0.27 ^ABCD;a^	5.3 ± 0.10 ^CDE;a^	7.5 ± 0.19 ^A;a^	5.4 ± 0.13 ^BCDE;a^	6.3 ± 1.44 ^ABCD;a^	7.4 ± 0.38 ^AB;a^
aristolene	1452	-	-	-	0.1 ± 0.05	-	-	-	-	-	-	-
*(E)*-β-farnesene	1458	0.3 ± 0.04	1.2 ± 0.07	1.4 ± 0.37	1.7 ± 0.19	1.1 ± 0.05	0.4 ± 0.01	0.1 ± 0.07	0.1 ± 0.09	0.3 ± 0.03	0.8 ± 0.19	0.1 ± 0.06
*allo*aromadendrene	1460	0.4 ± 0.07	0.9 ± 0.00	0.6 ± 0.16	0.7 ± 0.07	0.9 ± 0.01	0.4 ± 0.03	0.3 ± 0.03	0.5 ± 0.01	0.4 ± 0.02	0.5 ± 0.09	0.2 ± 0.01
4,5-di-*epi*-aristolochene	1468	-	-	-	-	-	-	-	-	-	-	-
γ-gurjunene	1469	-	-	-	-	-	-	-	-	-	-	-
β-chamigrene	1476	-	-	-	0.1 ± 0.07	-	0.1 ± 0.03	-	-	-	-	-
γ-muurolene	1477	0.3 ± 0.02	-	0.2 ± 0.05	0.2 ± 0.04	-	0.1 ± 0.01	-	-	0.4 ± 0.09	0.2 ± 0.04	-
α-amorphene	1482	0.2 ± 0.02	0.2 ± 0.01	0.1 ± 0.02	0.2 ± 0.04	-	0.2 ± 0.01	0.1 ± 0.01	0.1 ± 0.01	0.1 ± 0.03	0.1 ± 0.03	-
γ-selinene	1483	0.5 ± 0.01	0.6 ± 0.04	0.5 ± 0.11	0.6 ± 0.10	0.4 ± 0.01	0.6 ± 0.01	0.5 ± 0.02	0.5 ± 0.03	0.5 ± 0.04	0.5 ± 0.11	0.4 ± 0.01
**β-selinene**	1486	1.3 ± 0.03 ^CDE;a^	1.5 ± 0.10 ^CDE;a^	2.1 ± 0.48 ^AB;a^	2.2 ± 0.30 ^A;a^	1.9 ± 0.02 ^ABC;a^	1.5 ± 0.08 ^BCDE;a^	1.2 ± 0.07 ^E;a^	1.4 ± 0.13 ^CDE;a^	1.8 ± 0.09 ^ABCD;a^	1.3 ± 0.31 ^CDE;a^	1.3 ± 0.05 ^DE;a^
δ-selinene	1491	0.2 ± 0.01	0.1 ± 0.01	-	0.2 ± 0.04	-	0.3 ± 0.03	0.2 ± 0.02	0.2 ± 0.01	0.1 ± 0.04	0.1 ± 0.06	0.1 ± 0.05
valencene	1493	0.3 ± 0.01	0.3 ± 0.03	0.2 ± 0.05	0.3 ± 0.04	0.2 ± 0.00	0.3 ± 0.02	0.3 ± 0.05	0.3 ± 0.02	0.2 ± 0.00	0.3 ± 0.06	0.2 ± 0.02
α-selinene	1495	1.2 ± 0.00	1.2 ± 0.04	1.7 ± 0.40	1.5 ± 0.20	0.9 ± 0.09	1.5 ± 0.06	1.0 ±0.20	1.1 ±0.07	1.5 ± 0.16	1.0 ± 0.26	1.0 ± 0.07
eremophilene	1499	-	-	-	-	-	-	-	0.1 ± 0.04	-	-	-
α-bulnesene	1505	-	-	-	0.1 ± 0.02	-	1.4 ± 0.04	0.2 ± 0.05	-	0.1 ± 0.03	0.1 ± 0.01	-
*epi*zonarene	1501	-	-	-	0.2 ± 0.04	-	-	0.2 ± 0.05	-	0.2 ± 0.03	0.2 ± 0.04	-
β-bisabolene	1509	0.1 ± 0.00	0.3 ± 0.02	0.3 ± 0.06	0.3 ± 0.04	0.2 ± 0.01	-	-	-	0.9 ± 0.11	0.4 ± 0.09	-
*(E,E)*-α-farnesene	1509	-	-	-	-	-	0.3 ± 0.01	0.4 ± 0.08	0.4 ± 0.06	-	-	0.4 ± 0.01
β-curcumene	1513	-	0.1 ± 0.01	0.2 ± 0.05	0.2 ± 0.03	-	-	-	-	-	-	-
*trans*-γ-cadinene	1513	0.5 ± 0.01	0.1 ± 0.00	0.3 ± 0.06	0.2 ± 0.01	-	0.1 ± 0.01	-	-	0.4 ± 0.03	0.2 ± 0.03	-
sesquicineole	1516	-	0.6 ± 0.03	-	-	-	-	-	-	-	-	-
7-*epi-*α-selinene	1517	0.4 ± 0.37	-	0.4 ± 0.05	0.6 ± 0.08	0.3 ± 0.01	-	-	-	-	-	-
*(Z)-*γ-bisabolene	1519	-	-	-	-	-	-	-	-	-	-	-
β-cadinene	1519	0.3 ± 0.01	0.2 ± 0.01	-	0.1 ± 0.14	-	0.2 ± 0.01	0.2 ± 0.02	0.2 ± 0.02	0.4 ± 0.05	0.3 ± 0.05	0.1 ± 0.05
*trans*-calamenene	1524	-	-	-	-	-	0.1 ± 0.05	-	-	-	-	-
δ-cadinene	1524	0.5 ± 0.03	0.4 ± 0.02	0.6 ± 0.06	0.5 ± 0.07	0.2 ± 0.02	0.2 ± 0.01	0.1 ± 0.01	0.2 ± 0.02	0.5 ± 0.10	0.3 ± 0.07	0.1 ± 0.01
**selina-3,7(11)-diene**	1530	5.2 ± 0.47 ^BC;a^	3.2 ± 0.05 ^DE;a^	2.1 ± 0.65 ^DEF;a^	3.8 ± 0.55 ^CD;a^	1.3 ± 0,13 ^F;a^	3.8 ± 0.26 ^CD;a^	3.4 ± 0.33 ^DE^	3.1 ± 0.20 ^DE;a^	8.6 ± 1.01 ^A;a^	5.6 ± 1.30 ^B;a^	1.9 ± 0.09 ^EF;a^
*(E)*-γ-bisabolene	1531	-	-	-	-	-	-	-	-	-	-	-
α-calacorene	1543	0.4 ± 0.05	-	0.2 ± 0.01	0.3 ± 0.03	-	0.4 ± 0.04	0.2 ± 0.01	0.2 ± 0.02	-	-	0.1 ± 0.01
*cis*-sesquisabinene hydrate	1545	-	-	0.2 ± 0.05	-	0.1 ± 0.11	-	-	-	-	-	-
elemol	1550	0.4 ± 0.04	0.4 ± 0.04	0.4± 0.09	0.4 ± 0.06	0.6 ± 0.09	0.2 ± 0.05	0.2 ± 0.04	0.2 ± 0.02	0.2 ± 0.00	0.3 ± 0.04	0.2 ± 0.02
guaia-3,9-diene	1556	-	-	0.1 ± 0.10	-	-	-	-	-	-	-	-
*(E)*-nerolidol	1564	0.6 ± 0.04	1.2 ± 0.04	0.9 ± 0.36	0.8 ± 0.07	0.7 ± 0.12	1.2 ± 0.13	0.3 ± 0.04	0.6 ± 0.12	0.9 ± 0.11	1.5 ± 0.27	0.6 ± 0.01
palustrol	1568	-	-	0.4 ± 0.15	-	-	-	-	-	-	-	-
caryophyllene alcohol	1570	-	-	-	-	-	-	-	-	-	-	-
spathulenol	1577	-	-	-	-	-	-	-	-	-	-	-
*trans*-sesquisabinene hydrate	1581	-	0.1 ± 0.05	0.2 ± 0.05	0.1 ± 0.06	-	-	-	-	-	-	-
**caryophyllene oxide**	1582	11.8 ± 1.18 ^BCD;b^	11.3 ± 0.85 ^BCD;b^	10.9 ± 3.38 ^BCDE;a^	13.9 ± 0.24 ^BC;a^	22.7 ± 0.45 ^A;a^	14.4 ± 1.22 ^B;a^	7.0 ± 1.06 ^E;b^	9.9 ± 0.78 ^CDE;a^	9.1 ± 0.74 ^DE;a^	12 ± 2.03 ^BCD;a^	9.2 ± 0.11 ^DE;b^
globulol	1583	-	-	-	-	-	-	-	-	-	-	-
*trans-(Z)*-α-bisabolene epoxide	1586	-	-	0.5 ± 0.16	-	-	-	-	-	-	-	-
*iso*aromadendrene epoxide	1589	0.5 ± 0.10	0.5 ± 0.02	-	0.5 ± 0.06	0.8 ± 0.01	0.7 ± 0.07	0.3 ± 0.01	0.5 ± 0.04	0.5 ± 0.07	0.5 ± 0.08	0.4 ± 0.01
*epi-*globulol	1590	-	-	-	-	0.1 ± 0.01	-	-	-	-	-	-
viridiflorol	1592	0.2 ± 0.03	0.3 ± 0.05	-	0.3 ± 0.04	0.8 ± 0.04	0.2 ± 0.04	0.1 ± 0.02	0.2 ± 0.00	0.3 ± 0.07	0.2 ± 0.04	-
guaiol	1596	-	-	0.3 ± 0.09	-	-	-	-	-	-	-	-
**humulene oxide II**	1608	4.2 ± 0.58 ^BCD;b^	4.1 ± 0.55 ^BCD;b^	4.8 ± 1.08 ^BC;a^	4.9 ± 0.19 ^BC;a^	9.3 ± 0.46 ^A;a^	5.2 ± 1.40 ^B;a^	2.4 ± 0.24 ^D;a^	3.6 ± 0.32 ^BCD;a^	3.4 ± 0.28 ^BCD;a^	4.0 ± 0.58 ^BCD;a^	3.1 ± 0.03 ^CD;b^
cedrenol	1610	0.6 ± 0.11	0.1 ± 0.07	-	0.4 ± 0.03	-	0.5 ± 0.25	0.2 ± 0.00	0.3 ± 0.02	0.3 ± 0.02	0.2 ± 0.02	-
humulane-1-6-dien-3-ol	1613	-	-	0.3 ± 0.03	-	-	-	-	-	-	-	-
**selin-6-en-4-ol**	1618	1.1 ± 0.02 ^CDE;a^	1.1 ± 0.23 ^CDE;a^	0.5 ± 0.17 ^E;b^	1.2 ± 0.08 ^CD;a^	0.6 ± 0.05 ^DE;b^	2.0 ± 0.55 ^A;a^	0.8 ± 0.23 ^CDE;b^	0.8 ± 0.11 ^CDE;b^	1.9 ± 0.06 ^AB;a^	1.3 ± 0.14 ^BC;a^	0.7 ± 0.11 ^DE;b^
13-*nor*-valenc-1(10)-en-11-one	1629	-	0.2 ± 0.01	0.4 ± 0.06	0.5 ± 0.05	-	-	-	-	1.0 ± 0.04	0.3 ± 0.34	0.2 ± 0.01
1-*epi*-cubenol	1627	0.4 ± 0.10	0.3 ± 0.07	0.4 ± 0.06	0.3 ± 0.26	0.5 ± 0.06	0.5 ± 0.10	0.2 ± 0.04	0.2 ± 0.07	0.2 ± 0.00	0.4 ± 0.04	0.2 ± 0.01
γ-eudesmol	1631	-	-	-	-	-	-	-	-	-	-	-
**caryophylla-4(14),8(15)-dien-5-ol (unidentified isomer 1)**	1633	0.7 ± 0.69 ^F;b^	2.7 ± 0.46 ^AB;b^	2.5 ± 0.25 ^ABC;b^	2.6 ± 0.19 ^AB;a^	2.7 ± 0.27 ^AB;b^	3.0 ± 0.60 ^A;a^	1.4 ± 0.15 ^DEF;a^	0.9 ± 0.10 ^EF;b^	1.6 ± 0.06 ^CDEF;b^	2.3 ± 0.13 ^ABCD;b^	1.7 ± 0.09 ^BCDE;b^
**caryophylla-4(14),8(15)-dien-5-ol (unidentified isomer 2)**	1633	2.1 ± 0.21 ^ABC;b^	2.6 ± 0.54 ^AB;b^	2.8 ± 0.30 ^A;a^	2.6 ± 0.16 ^AB;b^	2.7 ± 0.31 ^A;b^	1.8 ± 0.44 ^BCD;a^	1.2 ± 0.09 ^D;b^	1.3 ± 0.04 ^D;b^	1.2 ± 0.03 ^D;b^	2.2 ± 0.12 ^ABC;b^	1.6 ± 0.07 ^CD;b^
T-cadinol	1641	-	-	0.3 ± 0.05	-	-	-	-	-	-	-	-
cubenol	1641	-	-	-	-	-	-	-	-	0.4 ± 0.02	-	-
β-eudesmol	1649	0.2 ± 0.08	0.1 ± 0.00	-	0.2 ± 0.08	-	0.3 ± 0.09	-	-	-	0.2 ± 0.00	-
α-eudesmol	1653	-	-	-	-	-	-	-	-	-	-	-
*neo*intermedeol	1655	-	-	0.3 ± 0.06	0.1 ± 0.11	0.3 ± 0.02	1.2 ± 0.00	-	-	0.2 ± 0.03	-	0.2 ± 0.00
bulnesol	1668	-	-	-	-	-	-	-	-	-	-	-
**14-hydroxy-9-*epi-(E)*-caryophyllene**	1670	3.8 ± 0.50 ^C;b^	2.9 ± 0.56 ^C;b^	5.8 ± 0.62 ^AB;a^	5.4 ± 0.39 ^B;b^	7.0 ± 0.53 ^A;b^	5.5 ± 0.19 ^B;b^	2.5 ± 0.12 ^C;b^	3.0 ± 0.28 ^C;b^	3.6 ± 0.81 ^C;b^	3.6 ± 0.40 ^C;b^	2.7 ± 0.21 ^C;b^
ylangenal	1675	-	1.8 ± 1.66	-	-	0.2 ± 0.02	0.2 ± 0.05	-	-	-	0.1 ± 0.06	-
aromadendrene epoxide II	1680	0.2 ± 0.03	0.2 ± 0.03	0.2 ± 0.05	0.2 ± 0.02	0.1 ± 0.01	0.1 ± 0.12	-	0.2 ± 0.01	0.1 ± 0.09	0.1 ± 0.06	0.1 ± 0.02
**α-bisabolol**	1685	0.4 ± 0.06 ^E;a^	1.7 ± 0.36 ^BC;b^	0.8 ± 0.12 ^DE;a^	0.4 ± 0.04 ^E;b^	0.3 ± 0.03 ^E;a^	1.2 ± 0.06 ^CD;a^	1.1 ± 0.13 ^CD;a^	0.6 ± 0.07 ^DE;b^	5.2 ± 0.49 ^A;a^	2.2 ± 0.36 ^B;b^	1.1 ± 0.04 ^CD;a^
cedr-8-en-13-ol	1688	-	-	-	-	-	-	-	-	-	-	-
juniper camphor	1694	1.0 ± 0.06	1.0 ± 0.24	0.4 ± 0.07	0.8 ± 0.08	0.5 ± 0.02	1.8 ± 0.03	0.9 ± 0.06	0.7 ± 0.07	1.0 ± 0.05	0.9 ± 0.18	0.7 ± 0.02
(Z)-α-*trans*-bergamotol	1700	-	-	-	-	-	0.2 ± 0.02	-	-	-	-	-
α-phellandrene dimer	1801	-	-	0.1 ± 0.02	-	-	-	-	-	-	-	-
nootkatone	1803	-	-	-	-	0.2 ± 0.01	0.2 ± 0.01	-	-	-	-	-
*(E,E)*-farnesyl acetate	1843	-	-	0.2 ± 0.02	-	-	-	-	-	-	-	-
**hexahydrofarnesylacetone**	1845	0.6 ± 0.11 ^D;a^	0.6 ± 0.15 ^D;b^	1.7 ± 0.24 ^BC;a^	1.0 ± 0.21 ^D;a^	1.9 ± 0.11 ^B;a^	2.3 ± 0.11 ^A;a^	0.8 ± 0.14 ^D;b^	1.5 ± 0.05 ^C;b^	0.7 ± 0.03 ^D;b^	0.7 ± 0.13 ^D;a^	0.7 ± 0.01 ^D;b^
1-hexadecanol	1877	-	-	-	-	-	-	-	0.1 ± 0.07	0.1 ± 0.05	-	0.1 ± 0.05
*(E,E)*-farnesyl acetone	1918	-	-	0.6 ± 0.12	-	0.6 ± 0.03	0.2 ± 0.06	-	-	-	0.1 ± 0.01	-
*m-*camphorene	1952	0.1 ± 0.01	-	0.2 ± 0.06	0.1 ± 0.01	-	0.1 ± 0.02	0.3 ± 0.08	0.3 ± 0.03	0.1 ± 0.01	-	0.3 ± 0.00
*p-*camphorene	1986	0.2 ± 0.10	0.1 ± 0.06	0.5 ± 0.07	0.4 ± 0.07	-	-	0.6 ± 0.11	0.6 ± 0.09	-	-	0.6 ± 0.00
ethyl hexadecanoate	2000	-	-	0.1 ± 0.07	-	-	-	-	-	-	-	-
**phytol**	2112	0.5 ± 0.14 ^CD;b^	0.4 ± 0.18 ^D;b^	0.8 ± 0.27 ^BCD;b^	0.4 ± 0.05 ^D;b^	3.2 ± 0.11 ^A;a^	2.7 ± 0.04 ^A;b^	0.9 ± 0.06 ^BC;a^	0.7 ± 0.19 ^BCD;b^	1.0 ± 0.07 ^B;b^	0.8 ± 0.23 ^BCD;a^	0.9 ± 0.11 ^BC;a^
**cannabidiol**	2369	23.2 ± 6.13 ^BCDE;a^	21.8 ± 0.66 ^BCDE;a^	28.2 ± 1.37 ^ABCD;b^	20.9 ± 3.94 ^CDE;a^	16.4 ± 0.36 ^DE;a^	14.5 ± 1.27 ^E;b^	35.9 ± 4.68 ^A;b^	29.6 ± 2.31 ^ABC;a^	22.2 ± 2.61 ^BCDE;a^	21.3 ± 10.13 ^CDE;a^	33.8 ± 0.98 ^AB;a^
cannabichromene	2373	0.7 ± 0.36	0.7 ± 0.23	0.4 ± 0.23	0.7 ± 0.25	0.7 ± 0.11	0.4 ± 0.14	1.2 ± 0.39	1.1 ± 0.11	0.7 ± 0.12	0.5 ± 0.29	1.1 ± 0.07
Δ^9^-tetrahydrocannabinol	2468	0.3 ± 0.09	0.4 ± 0.06	0.5 ± 0.01	0.2 ± 0.03	0.2 ± 0.00	0.2 ± 0.04	0.6 ± 0.16	0.5 ± 0.04	0.3 ± 0.08	0.3 ± 0.22	0.6 ± 0.01
Total identified (%)		94.5 ± 0.66	97.7 ± 0.08	98.1 ± 0.63	97.4 ± 0.09	99.4 ± 0.08	92.9 ± 1.03	96.9 ± 0.34	97.2 ± 0.25	95.8 ± 0.86	96.1 ± 1.06	97.2 ± 0.23
Monoterpene hydrocarbons		4.3 ± 0.71 ^BCD;a^	4.1 ± 1.57 ^BCD;a^	1.8 ± 0.52 ^DE;a^	2.0 ± 0.36 ^DE;a^	4.0 ± 0.60 ^BCD;a^	1.2 ± 0.15 ^E;a^	7.5 ± 0.42 ^A;a^	5.7 ± 2.07 ^AB;a^	3.7 ± 0.01 ^BCDE;a^	2.5 ± 0.85 ^CDE;a^	4.7 ± 0.35 ^BC;a^
Oxygenated monoterpenes		0.5 ± 0.10 ^DE;a^	0.9 ± 0.34 ^D;b^	0.3 ± 0.06 ^E;a^	0.2 ± 0.05 ^E;a^	0.5 ± 0.07 ^DE;a^	0.9 ± 0.15 ^D;a^	1.8 ± 0.20 ^BC;a^	2.3 ± 0.30 ^A;a^	1.4 ± 0.12 ^C;a^	0.6 ± 0.06 ^DE;a^	2.0 ± 0.10 ^AB;a^
Sesquiterpene hydrocarbons		35.5 ± 2.88 ^A;a^	35.6 ± 2.00 ^A;a^	29.6 ± 6.37 ^AB;a^	35.8 ± 3.03 ^A;a^	21.3 ± 0.88 ^B,a^	29.7 ± 3.84 ^AB;a^	28.5 ± 1.67 ^AB;a^	31.8 ± 3.45 ^AB;a^	34.5 ± 2.15 ^A;a^	36.9 ± 8.42 ^A;a^	29.6 ± 1.31 ^AB;a^
Oxygenated sesquiterpenes		28.2 ± 2.24 ^CD;b^	33.1 ± 4.36 ^BC;b^	33.2 ± 7.07 ^BC;a^	35.6 ± 1.25 ^BC;a^	50.3 ± 0.95 ^A;b^	40.3 ± 4.64 ^B;a^	18.6 ± 2.13 ^E;b^	22.9 ± 1.59 ^DE;b^	30.9 ± 0.22 ^CD;b^	32.3 ± 2.09 ^BC;b^	22.9 ± 0.47 ^DE;b^
Diterpene hydrocarbons		0.4 ± 0.11 ^BC;a^	0.1 ± 0.06 ^D;a^	0.9 ± 0.14 ^A;a^	0.5 ± 0.08 ^B;a^	- ^D^	0.1 ± 0.02 ^CD;a^	1.0 ± 0.19 ^A;a^	0.8 ± 0.12 ^A;a^	0.1 ± 0.01 ^CD;a^	- ^D^	0.9 ± 0.00^A;a^
Oxygenated diterpenes		0.5 ± 0.14 ^CD;b^	0.4 ± 0.18 ^D;b^	0.8 ± 0.27 ^BCD;b^	0.4 ± 0.05 ^D;b^	3.2 ± 0.11 ^A;a^	2.7 ± 0.04 ^A;b^	0.9 ± 0.06 ^BC;a^	0.7 ± 0.19 ^BCD;b^	1.0 ± 0.07 ^B;b^	0.8 ± 0.23 ^BCD;a^	0.9 ± 0.11 ^BC;a^
Phenylpropanoids		0.2 ± 0.06 ^AB;a^	0.1 ± 0.07 ^CD;a^	- ^D^	0.1 ± 0.03 ^BC;a^	0.3 ± 0.01 ^A;a^	- ^D^	- ^D^	- ^D^	- ^D^	0.2 ± 0.07 ^AB;a^	- ^D^
Cannabinoids		24.2 ± 6.57 ^BCDE;a^	22.8 ± 0.94 ^BCDE;a^	29.1 ± 1.61 ^ABCD;b^	21.8 ± 4.21 ^CDE;a^	17.4 ± 0.47 ^DE;a^	15.0 ± 1.45 ^E;b^	37.7 ± 5.23 ^A;a^	31.3 ± 2.45 ^ABC;a^	23.3 ± 2.80 ^BCDE;a^	22.1 ± 10.63 ^CDE;a^	35.5 ± 0.91 ^AB;a^
Apocarotenoids		0.6 ± 0.11 ^C;b^	0.6 ± 0.15 ^C;b^	2.4 ± 0.38 ^A;a^	1.0 ± 0.21 ^C;b^	2.5 ± 0.13 ^A;a^	2.5 ± 0.06 ^A;b^	0.8 ± 0.14 ^C;b^	1.5 ± 0.05 ^B;b^	0.7 ± 0.03 ^C;b^	0.8 ± 0.14 ^C;a^	0.7 ± 0.01 ^C;b^
Other non-terpene derivatives		0.1 ± 0.07 ^B;a^	- ^B^	0.1 ± 0.07 ^B;a^	- ^B^	0.1 ± 0.08 ^B;a^	0.4 ± 0.10 ^A;a^	0.1 ± 0.10 ^B;a^	0.2 ± 0.11 ^B;a^	0.1 ± 0.12 ^B;a^	- ^B^	0.1 ± 0.05 ^B;a^
Extraction yield (% *w*/*w*)		0.12 ± 0.01 ^CD;a^	0.23 ± 0.02 ^A;a^	0.12 ± 0.00 ^CD;a^	0.17 ± 0.01 ^ABC;a^	0.03 ± 0.00 ^E^	0.05 ± 0.01 ^DE;a^	0.13 ± 0.00 ^DE;a^	0.20 ± 0.02 ^AB;a^	0.20 ± 0.00 ^AB^	0.14 ± 0.04 ^BC;a^	0.16 ± 0.01 ^ABC;a^

^1^ Linear retention index on a HP 5-MS capillary column; ^2^ Not detected; ^3^ Compounds accounting for at least 1.000% of the dissimilarity rate (According to the SIMPER test, see Table 4) are evidenced in bold. For these compounds, for all chemical classes, and for the extraction yield, different superscript uppercase letters (A–F) indicate statistically significant differences between the each variety; superscript lowercase letters (a,b) indicate statistically significant differences among the cultivar withdrawn on different years (see Table 2 for 2019 samples). The statistical significance of the relative abundances was established by the Tukey’s *post-hoc* test, with *p* ≤ 0.05.

**Table 4:** Two-way ANOVA performed on: (i) SIMPER-selected compounds; (ii) all detected chemical classes; (iii) EO extraction yield.

	Genotype (G)	Year (Y)	Genotype × Year (G × Y)
**SIMPER-selected compounds (≥1% dissimilarity contribution)**			
cannabidiol	***	n.s.	***
β-caryophyllene	***	***	**
14-hydroxy-9-*epi-(E)-*caryophyllene	***	***	***
caryophyllene oxide	***	**	***
caryophylla-4(14),8(15)-dien-5-ol (unidentified isomer 1)	***	***	***
α-humulene	***	***	***
caryophylla-4(14),8(15)-dien-5-ol (unidentified isomer 2)	***	***	***
humulene oxide II	***	**	***
selina-3,7(11)-diene	***	***	***
α-bisabolol	***	***	***
phytol	***	***	***
α-pinene	***	***	**
selin-6-en-4-ol	***	***	**
myrcene	***	***	***
hexahydrofarnesylacetone	***	***	***
β-selinene	***	***	***
**All detected chemical classes**			
Monoterpene hydrocarbons	***	***	***
Oxygenated monoterpenes	***	***	***
Sesquiterpene hydrocarbons	***	***	***
Oxygenated sesquiterpenes	***	***	***
Diterpene hydrocarbons	***	***	***
Oxygenated diterpenes	***	***	***
Phenylpropanoids	***	**	**
Cannabinoids	***	n.s.	***
Apocarotenoids	***	***	***
Other non-terpene derivatives	***	**	**
**Extraction yield (% *w/w*)**			
EO hydrodistillation yield (% *w/w*)	***	***	***

LSR, ** *p* < 0.01; *** *p* < 0.0001. n.s. = not significative.

**Table 5:** Weather parameters during field trials at Rovigo. Rainfall is expressed as millimeters per day (mm/day), Relative Humidity (RH) is expressed as percentage.

Month	First Season (2019)					Second Season (2020)				
	Min Temp	Max Temp.	Av. Temp.	Rainfall (mm)	RH (%)	Min Temp	Max Temp.	Av. Temp.	Rainfall	RH
January	−1.1	6.3	2.6	16.6	81.1	0.0	8.0	3.5	2.0	87.6
February	0.9	12.2	6.6	26.6	76.3	2.3	13.2	7.3	2.8	73.0
March	3.3	17.0	9.9	6.4	66.7	4.4	14.2	9.2	30.4	70.1
April	11.7	12.1	16.9	86.4	73.7	7.1	21.1	14.0	9.6	59.4
May	11.3	20.0	15.1	151.2	78.9	12.7	24.6	18.5	13.0	67.5
June	18.8	31.7	25.3	5.6	65.5	16.1	27.7	21.6	77.4	69.5
July	19.7	31.7	25.3	66	69.0	18.3	30.3	24.1	76.8	69.6
August	19.9	30.7	24.8	46.6	73.4	19.0	30.7	24.4	91.8	73.0
September	15.1	25.7	19.8	71.8	73.7	14.9	26.6	20.3	19.6	70.7
October	11.9	20.8	15.8	29.4	82.7	8.9	18.4	13.1	70.4	80.6
November	7.6	13.5	10.4	172.6	89.2	7.8	12.5	8.4	11.0	78.1
December	2.2	9.0	5.4	82	87.6	3.4	8.0	5.6	73.0	92.0

## Data Availability

Data is contained within the article.

## Sample Availability

Samples of the compounds are available from the authors.
